# The Loss of Mitochondrial Quality Control in Diabetic Kidney Disease

**DOI:** 10.3389/fcell.2021.706832

**Published:** 2021-08-05

**Authors:** Wenni Dai, Hengcheng Lu, Yinyin Chen, Danyi Yang, Lin Sun, Liyu He

**Affiliations:** ^1^Hunan Key Laboratory of Kidney Disease and Blood Purification, Department of Nephrology, The Second Xiangya Hospital of Central South University, Changsha, China; ^2^Department of Nephrology, Hunan Provincial People’s Hospital, The First Affiliated Hospital of Hunan Normal University, Changsha, China

**Keywords:** diabetic kidney disease, mitochondrial quality control, oxidative stress, mitochondrial dynamics, mitophagy, mitochondrial biogenesis, mitochondrial protein quality control

## Abstract

Diabetic kidney disease (DKD) is the predominant complication of diabetes mellitus (DM) and the leading cause of chronic kidney disease and end-stage renal disease worldwide, which are major risk factors for death. The pathogenesis of DKD is very complicated, including inflammation, autophagy impairment, oxidative stress, and so on. Recently, accumulating evidence suggests that the loss of mitochondrial quality control exerts critical roles in the progression of DKD. Mitochondria are essential for eukaryotic cell viability but are extremely vulnerable to damage. The mechanisms of mitochondrial quality control act at the molecular level and the organelle level, including mitochondrial dynamics (fusion and fission), mitophagy, mitochondrial biogenesis, and mitochondrial protein quality control. In this review, we summarize current knowledge of the role of disturbances in mitochondrial quality control in the pathogenesis of DKD and provide potential insights to explore how to delay the onset and development of DKD.

## Introduction

Mitochondria are one of the important intracellular organelles that participate in a broad array of functions in mammals (Dimmer and Scorrano, 2006). Mitochondria not only produce energy for cells through oxidative phosphorylation to produce ATP but also participate in the maintenance of cellular calcium and redox homeostasis, the generation of reactive oxygen species (ROS), the regulation of various catabolic and anabolic processes, which is of great significance for maintaining the homeostasis of the internal environment of cells and the life activities of bodies (Nunnari and Suomalainen, 2012). When the components of the proteins, lipids, and DNA in the organelles are damaged, the steady-state of the mitochondria is maintained by many mechanisms to maintain the integrity of the mitochondrial structure and function, which is commonly referred to as a mitochondrial quality control. The mechanisms of mitochondrial quality control act at the molecular level and the organelle level. The former includes mitochondrial protein quality control, such as the regulation of mitochondrial protease and molecular chaperone, while the latter includes mitochondrial dynamics (fusion and fission), mitophagy, mitochondrial biogenesis (Tang et al., 2020). If these mechanisms go out of control, it will lead to a range of diseases, such as metabolic, cardiovascular (Fan et al., 2020), neurodegenerative diseases (Gao and Zhang, 2018), and cancer (Zong et al., 2016).

The kidney is a highly metabolic organ (Soltoff, 1986) and the most important target of microvascular damage in diabetes (Forbes and Cooper, 2013), having the second-highest resting metabolic rates according to a study (Wang et al., 2010), whose function is tightly related to mitochondria energy production. Given that the kidney requires an abundance of mitochondria to provide sufficient energy to work, it is vital for the maintenance of mitochondrial homeostasis. The loss of mitochondrial quality control exerts an important role in the progression of diabetic kidney disease (DKD) because it destroys the homeostasis of mitochondria, thus damaging kidney. High levels of blood glucose can lead to the overgeneration of ROS, causing oxidative damage to mitochondrial lipids, DNA, and proteins, making mitochondria further prone to ROS production, which forms a vicious cycle. In addition, strategies targeting mitochondrial quality control mechanisms may be promising therapies to prevent and treat DKD. Therefore, this review is designed to clarify the role of disturbances in mitochondrial quality control in the pathogenesis of DKD and provide potential therapeutic strategies through comprehensive medical articles.

## Mitochondrial Quality Control

Coping with internal and external stresses, the mechanism of mitochondrial quality control is critical to avoid mitochondrial DNA mutations and protein misfolding because mitochondria are exposed to high amounts of ROS. The normal function of mitochondria requires the synchronization of gene expression in the nucleus and mitochondria (Anderson et al., 1981). Moreover, it is also necessary that mitochondrial proteins import into the organelle from the cytosol because a very limited number of mitochondrial proteins and peptides are synthesized inside the organelle. Therefore, mitochondria have developed a variety of elaborate quality control systems to ensure the formation of a healthy and functional mitochondrial network. Physiologically, mitochondrial removal and replenishment reach a balance to maintain cell homeostasis (Pickles et al., 2018). Mitochondrial dynamics (fusion and fission), mitophagy, mitochondrial biogenesis, and mitochondrial protein quality control are interlinked that play pivotal roles in mitochondrial quality control for cellular homeostasis by maintaining the relative stability of mitochondrial morphology, quantity, and quality (Tang et al., 2020).

Mitochondrial fission and fusion events separate damaged mitochondria, meet the energy demands of cells and provide a mitochondrial quality control mechanism (Forbes and Thorburn, 2018). Mitochondrial removal is accomplished by mitophagy, which contributes to the degradation and reuse of damaged mitochondria. The process of mitochondrial biogenesis consists of highly regulated transcriptional events, new proteins and/or lipids synthesis/assembly, and replication of mtDNA, which produces new and functional mitochondria. In addition, the mitochondrial proteome displays high plasticity to allow the adaptation of mitochondrial function to cellular requirements and mitochondrial protein quality control is also important. If these mechanisms are defective, then causing obstacles to the clearance of damaged mitochondria and resulting in abnormal morphology and dysfunction of mitochondria, a variety of human disorders will happen, such as neurodegenerative disease, cancer, DKD, and so on.

In the proximal tubules of DKD had observed that the morphology of mitochondria changed, mitochondria fragmented into short rods or spheres, and had cristolysis. The study indicates that mitochondrial damage could be the hallmark of DKD patients (Jiang et al., 2019). The accumulation of damaged mitochondrial and fragmented mitochondria resulted in the overproduction of ROS and loss of mitochondrial membrane potential for mitochondrial dysfunction.

## Diabetic Kidney Disease

According to a report, the population of global diabetes mellitus (DM) patients is 463 million people in 2019, however, the incidence of DM is increasing year by year, which has become a global public health problem threatening human life and health (Saeedi et al., 2019). In either type1 diabetes mellitus (T1DM) or type 2 diabetes mellitus (T2DM), 30–40% of patients develop renal damage and approximately 5% of patients with T2DM have DKD when they are diagnosed with DM (Wang et al., 2008). DKD is one of the most predominant microvascular complications of DM and is the major cause of chronic kidney disease and end-stage renal disease, which contributes to high mortality (Forbes and Cooper, 2013; Thomas et al., 2015). Therefore, clarifying the pathogenesis of DKD have great significance for the diagnosis and treatment of DKD that we will focus on.

It is believed that DKD is caused by multiple factors, such as high glucose, oxidative stress, inflammatory response, genetic background, and environmental factors (Thomas et al., 2015). Glucose metabolism disorder is the basic pathophysiological change of DM and the basic factor of the occurrence and development of DKD (Nathan et al., 2013). Oxidative stress caused by sustained high blood sugar is the central part of DKD, which can lead to the release of excessive ROS, a large number of cytokines, inflammatory mediators, and activation of protein kinase C and other downstream signaling pathways, thus further damage the cells of the kidney, such as glomerular endothelial cells, mesangial cells, renal tubular epithelial cells and so on. Overproduction of ROS is a toxic byproduct of oxidative phosphorylation, which exceeds the normal ability of the body to remove oxygen, thus causing tissues injury and organs failure, especially mitochondrial damage (Wallace, 2005). ATP generation is increased early in DKD (Miyamoto et al., 2016) but later declines (Coughlan et al., 2016). In diabetic patients, changing the source of metabolic fuel to meet ATP needs can lead to increased oxygen consumption, which can lead to hypoxia in the kidneys (Hansell et al., 2013).

Diabetic kidney disease is a clinical syndrome having changes in the renal structure and function, involving progressive stages of glomerular ultrafiltration, microalbuminuria, persistent albuminuria, and decreased glomerular filtration rate (GFR), culminating in dialysis (Adler et al., 2003). Clinical features can help differentiate DKD from other kidney diseases. The onset of proteinuria and progression of DKD is gradual and the duration of diabetes ≥12 years is the best predictor of DKD alone, always accompanied by retinopathy (Doshi and Friedman, 2017). Current treatment strategies include blood glucose control, blood pressure control, a healthy lifestyle, and drugs. Some lifestyle interventions recommended for patients with DKD include more daily exercise, weight loss, smoking cessation, and sodium limitation (Thomas et al., 2015).

## Mitochondrial Quality Control and DKD

### Mitochondrial Dynamics and DKD

Mitochondria are double-membrane systems, which are divided into the outer mitochondrial membrane (OMM), intermembrane space (IMS), inner mitochondrial membrane (IMM), and the matrix from the outside to the inside (Frey and Mannella, 2000), observed by electron microscopy (EM). In the past, mitochondria were primarily considered as static and isolated structures. With the development of technology, the dated concept has dramatically changed by mitochondria being dynamic and highly motile organelles that move within the cell and frequently undergo coordinated cycles of fission and fusion for maintenance of mitochondrial turnover and cellular network balance (Lee and Yoon, 2016).

Mitochondrial fission and fusion events can redistribute content throughout the mitochondrial network, enabling the cell to adjust to the changing local needs (Hoppins et al., 2007). Fusion, the combining of two mitochondria, allows extensive exchanges and a mixture of mitochondrial mtDNA and protein (Twig et al., 2008; Chan, 2012). Fission, the splitting of mitochondrion into two, is discovered two functionally and mechanistically distinct types which include midzone and peripheral fissions. Different type of fission forms different daughter mitochondria. Division at the midzone enables biogenesis of new healthy mitochondria, while division at the periphery forms defective daughter mitochondria which can be removed and recycled via the mitophagy machinery (Kleele et al., 2021). If the depolarized mitochondria are not eliminated, Ca^2+^ and cytochrome c (Cyt c) may be released into the cytosol at high levels to facilitate cellular apoptosis (Youle and van der Bliek, 2012). Mitochondrial dynamics means the balance between fission and fusion (Liesa et al., 2009). Moreover, the harmonization of mitochondrial dynamics guarantees normal mitochondrial structure and morphology, which are imperative to mitochondrial quality control mechanisms for cellular homeostasis (van der Bliek et al., 2013).

Mitochondrial fission and fusion require a suite of proteins, which belong to the members of the GTPases family (Okamoto and Shaw, 2005). Fusion event is regulated by Mitofusins (MFN1 and MFN2) (Rojo et al., 2002) and optic atrophy protein 1 (OPA1), which mediate the fusion of the outer and IMMs, respectively (Rojo et al., 2002). The process of fusion contributes to mitochondrial elongation physiologically and maintains cellular phosphorylation levels. In contrast to fusion, fission mainly involves dynamin-1-like protein (DRP1) and mitochondrial fission protein 1 (FIS1) (Wang et al., 2012). The balance of mitochondrial fission and fusion contributes to cellular homeostasis. If fission is not controlled and balanced by fusion, the network becomes too fragmented (Bartolák-Suki et al., 2017). The disruption of these events prevents the elimination of damaged mitochondria and leads to the down-regulation of ATP production (Forbes and Thorburn, 2018).

The current body of evidence highlights the disorder of mitochondrial dynamics as an important mechanism of the progression of DKD. A study revealed that changes in mitochondrial dynamics precede the development of albuminuria and renal histological changes in DKD (Coughlan et al., 2016). Similar results have also shown that mitochondrial fragmentation was specifically presented within proximal tubule epithelial cells (PTECs) in DKD patients (Jiang et al., 2019). However, the process of fusion might be required to protect against kidney damage in diabetes (Nunnari and Suomalainen, 2012). MFN2 is found to regulate mitochondrial morphology and signaling. In a rat model of DKD, it has shown impaired MFN2 expression (Tang et al., 2012; Zhan et al., 2015). Strategies to knock down the expression of MFN2 lead to the lack of coenzyme Q10 and the decrease of mitochondrial production (Mourier et al., 2015). A study demonstrates that the overexpression of MFN2 can alleviate the pathological changes in STZ-induced diabetic rat kidneys via inhibiting activation of P38 and the accumulation of ROS (Tang et al., 2012). Therefore, MFN2 might provide a potential target for the treatment of DKD. In addition, MFN2 plays a role in mitophagy which is discussed in the mitophagy and DKD part.

Mitochondrial fission is a multi-step process, which is typically regulated by DRP1. DRP1 binds to its receptors on OMM and assembles a multimeric complex, eventually dividing mitochondrial tubules into membrane scission (Smirnova et al., 2001). While calcium influx is considered to be the regulation of mitochondrial intramembrane constriction. Promoting DRP1 expression contributes to mitochondrial fission (Smirnova et al., 2001; Hu et al., 2017). There is evidence that excessive mitochondrial fission is one of the characteristic features of mitochondrial dysfunction in diabetic kidneys (Wang et al., 2012). The processes of fission and fragmentation of mitochondria are commonly considered to be harmful in DKD (Nunnari and Suomalainen, 2012). Experiments in rodents (Coughlan et al., 2016) and humans (Zhan et al., 2018) have shown that perturbations in mitochondrial dynamics led to the renal tubular injury in DKD. Increased mitochondrial fission is associated with changes in markers of kidney injury, such as GFR and albuminuria (Zhan et al., 2013; Eid et al., 2019). In a genetically modified mouse model of T2DM, *Drp1* was selectively deleted in podocytes, which can ameliorate mitochondrial dysfunction and protect against the progression of DKD (Ayanga et al., 2016).

Furthermore, using the pharmacologic inhibitor of DRP1, Mdivi1, could be sufficient to block mitochondrial fission and rescued key pathologic features of DKD in mice (Ayanga et al., 2016). Berberine (BBR), a kind of Chinese herbal medicine, could significantly protect glomerulus and podocytes via alleviating mitochondrial fission, serving as a novel therapeutic strategy to treat DKD (Qin et al., 2019). In addition, Empagliflozin could reduce mitochondrial fission to improve the DKD via Adenosine monophosphate-activated protein kinase (AMPK)/SP1/Phosphoglycerate mutase family member 5 (PGAM5) pathway (Liu et al., 2020). Moreover, a research demonstrated that HIF-1α facilitated mitochondrial fusion and inhibited mitochondrial fission via the HO−1 pathway, then ameliorates tubular injury in DKD (Jiang et al., 2020).

### Mitophagy and DKD

Autophagy (meaning self-eating), a fundamental process, is a lysosome-dependent intracellular degradation system required for various normal physiological processes (Mizushima et al., 2008). The aim of autophagy is not only the degradation of cytosolic components and organelles but also acts as a dynamic circulatory system to regulate their number and maintain quality control for cellular renovation and homeostasis (Mizushima and Komatsu, 2011). Autophagy is classified into three distinct types, containing macroautophagy (Feng et al., 2014), microautophagy (Oku and Sakai, 2018), and chaperone-mediated autophagy (CMA) (Kaushik and Cuervo, 2018). Macroautophagy (Feng et al., 2014) is considered as the most prevalent form of autophagy, a process during which cargo that is sequestered within cytosolic double-membrane vesicles termed autophagosomes is delivered to lysosomes through vesicular fusion and is degraded by lysosomes. After degradation, the resulting macromolecular constituents are reused and recycled in the cytosol (Yorimitsu and Klionsky, 2005).

Mitophagy (Ashrafi and Schwarz, 2013),a selective autophagy of mitochondria, occurs through both macroautophagy and microautophagy processes. Mitophagy acts as a critical component of mitochondrial quality control mechanism that mediates clearance of damaged and unwanted mitochondria, then renew components (Ashrafi and Schwarz, 2013; Pickles et al., 2018). Mitophagy is a double-edged sword, moderate mitophagy can remove damaged mitochondria and reduce cell death and tissue damage while mitophagy disorder or excessive mitophagy can cause cell energy metabolism disorder and aggravate cell apoptosis.

**FIGURE 1:**
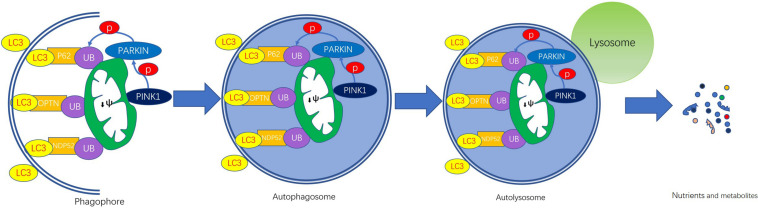
PINK1/Parkin-mediated mitophagy. The steps of mitophagy include the formation of the phagophore, autophagosome, autolysosome, and degradation of mitochondria. Damaged mitochondria that have lost their membrane potential (ψm) leads to PINK1 accumulation on the outer membrane surface. Then PINK1 recruits cytosolic Parkin, thereby phosphorylating. Activated Parkin contributes to the hyper-ubiquitination of OMM proteins, which are recognized by ubiquitin-binding adaptors, such as p62, NBR1, OPTN, and NDP52. These adaptor proteins induce the formation of autophagosomes by binging to LC3. The fusion of autophagosome and lysosome forms autolysosome in which enzymes are released to digest the contents and the nutrients and metabolites are recycled.

Mitophagy regulatory pathways (Hamacher-Brady and Brady, 2016) are classified as ubiquitin-dependent or -independent (Ashrafi and Schwarz, 2013; Khaminets et al., 2016). PTEN-induced putative kinase 1 (PINK1)-Parkin pathway regulates ubiquitin-dependent mitophagy (Narendra et al., 2008; Pickles et al., 2018) and receptor-mediated mitophagy pathway is ubiquitin-independent. The receptor in the ubiquitin-independent pathway is made up of Bnip3 (Hamacher-Brady et al., 2007; Zhu et al., 2013), its homolog Bnip3L/Nix (Schweers et al., 2007; Novak et al., 2010), FUNDC1 (Liu et al., 2012) and Bcl2L13 (Murakawa et al., 2015), which localize to the OMM. Mitophagy is mainly mediated by the PINK1-Parkin pathway in mammals (Figure 1). Next, we will introduce this pathway in detail.

Mitophagy can be mechanistically broken down into the following four steps: phagophore formation, autophagosome formation, autophagosome-lysosome fusion, and degradation of mitochondria (Youle and Narendra, 2011). With the help of LC3/ATG8, the isolation double-membrane elongates and closes, then forms an autophagosome engulfing the damaged mitochondrion separated from the mitochondrial network. In the end, the mitochondrial cargo is degraded by the lysosome. During the process, two genes are essential, encoding the PINK1 (Valente et al., 2004) and the cytosolic E3 ubiquitin ligase Parkin (Kitada et al., 1998), which are found to be mutated in Parkinson’s disease (PD).

PTEN-induced putative kinase 1 is not only a serine/threonine kinase but also a mitochondrial localized kinase with mitochondrial targeting sequences (MTSs) (Youle and Narendra, 2011). In normal mitochondria, PINK1 is imported to the IMM by several proteins. PINK1 MTS is cleaved by mitochondrial-processing protease (MPP) and PINK1 is cleaved by presenilin-associated rhomboid-like protease (PARL), then degraded (Meissner et al., 2011; Greene et al., 2012).

In damaged mitochondria, dissipation of membrane potential provides a signal to initiate the process of mitophagy because the defective mitochondria are unable to import and degrade PINK1 (Matsuda et al., 2010). A study demonstrated that PHB2 (prohibitin 2) can stabilize PINK1 in the surface of mitochondria via the PARL-PGAM5-PINK1 axis (Yan et al., 2020). In this manner, PINK1 accumulates on the outer membrane (TOM) of damaged mitochondria (Hasson et al., 2013; Sekine and Youle, 2018). Dependent on its kinase activity, PINK1 facilitates recruitment of Parkin from the cytosol to the PINK1-bound compartment (Lazarou et al., 2012) in the OMM, thereby phosphorylating Parkin, then inducing mitophagy (Kazlauskaite et al., 2014; Sekine and Youle, 2018). But the mechanism by which PINK1 recruits Parkin to damaged mitochondria is yet unknown. Once the activation, Parkin mediates ubiquitination of several outer membrane components and catalyzes the formation of poly-ubiquitin chains on OMM proteins that recruit adaptor proteins such as p62/SQSTRM1, optineurin (OPTN), NBR1, and so on (Lazarou et al., 2015; Harper et al., 2018). These adaptor proteins induce the formation of autophagosome by bind to LC3 proteins on phagophore and autophagosome membranes (Khaminets et al., 2016). Enzymes are released to digest the contents of the autophagosome, which are then excreted or redirected into cellular metabolism (Higgins and Coughlan, 2014). Interestingly, PINK1 can also phosphorylate ubiquitin (Ub) and poly-Ub chains on dysfunctional mitochondria, serving as an “eat me” signal for the autophagic machinery (Harper et al., 2018).

MFN2 not only plays an important role in mitochondria dynamics but is also closely related to mitophagy. As a mitochondrial receptor for Parkin in OMM, MFN2 is directly involved in the regulation of mitophagy. MFN2 is phosphorylated by PINK1 which can recruit Parkin. Then Parkin contributes to the ubiquitination of MFN2, thereby inhibiting mitochondrial fusion and leaving damaged mitochondria to exist alone (Chen and Dorn, 2013). Tubulointerstitial fibrosis is a common manifestation of late DKD (Tervaert et al., 2010). A study, utilizing 2 different models of kidney fibrosis, indicated that the renal expression of PINK1, MFN2, and Parkin is down-regulated during kidney fibrosis and implicates the relevance in the development of kidney fibrosis. The down-regulation of mitophagy regulators results in defective mitophagy, causes accumulation of abnormal mitochondria and higher ROS and rictor production which can promote the differentiation of macrophages, then leading to overproduction of extracellular matrix and kidney fibrosis (Bhatia et al., 2019). If therapy can up-regulate PINK1, MFN2, and Parkin, it will ease the progression of kidney fibrosis and protect against the advancement of CKD, such as DKD.

Mitophagy is closely related to mitochondrial dynamics. The balance in fission/fusion dynamics and mitophagy is crucial. The defective mitochondria are cleared by mitophagy. A study has shown that DRP1 and parkin played synergistic roles in mitochondrial homeostasis and survival. If deficiency DRP1 and parkin, mitochondrial degradation is further decreased (Kageyama et al., 2014). Mitochondrial dynamics and mitophagy have been linked to DKD. Specific mitochondrial fragmentation and the decreased expression of PINK1 and Parkin have been found in DKD (Xiao et al., 2017; Jiang et al., 2019). The impaired mitochondria increase, however, the process of mitophagy is blocked, the impaired mitochondria cannot be degraded and accumulate in cells, then release the amount of ROS, leading to the death of the renal cell. But a study demonstrated a different view of the mitophagy process of DKD, in which PINK1/Parkin-mediated mitophagy was abnormally activated in db/db mice (Liu et al., 2017). The different result may be that the DKD is under different stages. In the early stage of DKD, mitophagy is increased with the increase of defective mitochondria which is called compensation. In the late stage of DKD, mitophagy cannot demand the meet and is impaired, exhibiting a state of decompensation.

Multiple pathways are involved in mitophagy. In response to decreases in ATP, AMPK is activated to promote mitophagy. Under nutrient sufficiency, high mTOR activity prevents Ulk1 activation by phosphorylating Ulk1 Ser 757 to inhibit mitophagy (Kim et al., 2011). mTORC1 hyperactivation is a molecular signature of DKD (Gödel et al., 2011). HG causes a decrease in SIRT6 and dephosphorylation in AMPK. Fan et al. (2019) discovered that HG-induced a reduction in SIRT6 expression and p-AMPK down-regulation both *in vivo* and *in vitro* studies, accompanied by mitochondrial morphological abnormalities and impaired mitophagy. This study also found that SIRT6 overexpression mitigated mitochondrial dysfunction and podocyte damage induced by high glucose (Fan et al., 2019). Forkhead-box class O1 (FoxO1) has been reported to mediate PINK1 transcription and promote mitophagy in response to mitochondrial oxidative stress (Li et al., 2017). This insufficient mitophagy results in ROS overproduction and renal injury. Once mitophagy is activated, it can contribute to protect mitochondrial function, so as to improve the pathological state of DKD. For example, MitoQ can restore mitophagy to protect the kidney via regulating PINK1 and Parkin expression in diabetic mice (Xiao et al., 2017).

### Mitochondrial Biogenesis and DKD

Mitochondrial biogenesis is a multifaceted process involving lipid membrane and protein synthesis and replication of mtDNA, which produces new and functional mitochondria (Ventura-Clapier et al., 2008). This process is regulated by a range of transcriptional co-activators and co-repressors. Peroxisome proliferator-activated receptor-gamma coactivator-1 alpha (PGC-1α), the founding member of a family of transcriptional co-activators, is widely accepted as the “master regulator” of mitochondrial biogenesis by activating different transcription factors (Puigserver et al., 1998; Scarpulla, 2011). Low PGC-1α levels and decreased transcription of its gene targets damage mitochondrial biogenesis which has been linked to the onset and progression of multiple diseases. PGC-1α is regulated by CHOP, Smad3, and NF-kB negatively. The expression of PGC-1α is increased by NOS (Nisoli et al., 2003), SIRTs (Rodgers et al., 2005), TORCs (Wu et al., 2006), and AMPK (Hardie, 2007), which leads to an increase in nuclear respiratory factors (NRFs) (Puigserver et al., 1998), then improving mtDNA expression and mitochondrial proteins translation, thus promoting mitochondrial biogenesis. AMPK acts as an energy sensor of the cell and works as a key regulator of mitochondrial biogenesis, which is of major importance (Jornayvaz and Shulman, 2010).

In recent years, substantial attention has been focused on the process of mitochondrial biogenesis in DKD. Many lines of evidence indicate that mitochondrial biogenesis plays a beneficial role in DKD. PGC-1α is highly expressed in the proximal tubules where mitochondria are abundant (Portilla et al., 2002). Previous research reported that mRNA and protein expression of mitochondria-related protein PGC-1α decreased in HK-2 cells in HG ambiance (Yuan et al., 2018). Reduced levels of PGC-1α have been observed in diabetic rat kidneys (Guo et al., 2015). Knockout of *PGC-1*α in renal tubular cells results in significant mitochondrial damage (Svensson et al., 2016). *In vitro*, hyperglycemia induced the down-regulation of PGC-1α, which leads to mitochondrial biogenesis disorder, resulting in increased DRP1 expression, increased mitochondrial fragmentation, and impaired mitochondrial network structure. Given the essential role of PGC-1α in controlling mitochondrial biogenesis, it has been suggested as a pharmacological target to ameliorate renal mitochondrial dysfunction.

### Mitochondrial Protein Quality Control and DKD

To perform their multiple functions, mitochondria need a set of proteins to build the mitochondrial proteome, containing about 1,000–1,500 different proteins (Smith et al., 2012). About 99% of the mitochondrial proteins are encoded by nuclear genomes while the mitochondrial DNA encodes 13 proteins in humans. Owing to the majority of proteins encoded by nuclear genomes, these proteins are synthesized in the cytoplasmic ribosome and subsequently introduced into the mitochondria (Anderson et al., 1981). Healthy mitochondria depend on the integrity and homeostasis of the mitochondrial proteome. Cells use different cellular quality control systems to monitor the mitochondrial proteome. Mitochondrial molecular chaperones (Wiedemann and Pfanner, 2017), proteases (Quirós et al., 2015), the ubiquitin-proteasome system (UPS) (Nandi et al., 2006), mitochondrial unfolded protein response (UPR^mt^) (Quirós et al., 2015) are interlinked termed as mitochondrial protein quality control, which is helping correct protein folding, removing misfolded or aggregated proteins and eliminating dysfunctional mitochondria. Maintenance of this complex and adaptable mitochondrial proteome is of crucial importance to cell function. Dysregulation of mitochondrial protein homeostasis will lead to mitochondrial dysfunction and eventually cell death.

Precursor proteins are imported into the mitochondria via translocase of TOM and translocase of the inner membrane (TIM), which need to be in an unfolded state with the help of mitochondrial molecular chaperones, commonly known as heat shock protein 70 (Hsp70) (Wiedemann and Pfanner, 2017). Chaperones are also responsible for inhibiting the misfold of precursor proteins and ensuring correct protein import. In the early model of DKD, the overexpression of HSP27, HSP60, and HSP70 has been seen which can protect the kidney of cells (Barutta et al., 2008). Another study has been shown decreased HSP90 expression may mediate podocyte apoptosis (Kim et al., 2014). These findings suggest that the HSP family plays a momentous role in mitochondria.

Mitochondrial proteases (mitoproteases) are categorized into the resident mitoproteases and transient mitoproteas. Compared transient mitoproteas, resident mitoproteases, which conclude inner membrane-embedded AAA protease, matrix-embedded AAA protease, mitochondrial intermediate pre-sequence protease, matrix processing peptidase, lon protease, and so on, are well studied assisting in protein turnover and processing (Quirós et al., 2015). Lon protease is localized in the mitochondrial matrix, a study demonstrated that the Podocyte-specific deletion of Lon protease 1 induced mitochondria dysfunction and kidney injury (Gong et al., 2021).

Unfolded protein response is a stress response that activates gene transcription of nuclear-encoded mitochondrial chaperones and proteases to alleviate (Yoneda et al., 2004). Mitochondria have their proteolytic system, allowing them to degrade misfolded proteins that is important in mitochondrial function, integrity, and homeostasis (Quirós et al., 2015). The UPS is characterized by the strong dependence on Ub, mostly degrading single, unfolded polypeptides able to enter into the narrow channel of the proteasome (Nandi et al., 2006; Pohl and Dikic, 2019). The UPS regulates renal fibrosis and renal inflammation, which will develop specific new treatment options in renal disease (Meyer-Schwesinger, 2019). To date, evidence for the loss of mitochondrial protein quality control works out in DKD. However, the detailed mechanism is unclear and needs further study in the future.

## Therapeutic Outlook

At present, the basic intervention methods of DKD are strict control of blood sugar and blood pressure (BP) (Doshi and Friedman, 2017). Evidence suggests that tight glucose control and BP control significantly reduce the occurrence of DKD incidence (1998) (Berl et al., 2005). A healthy lifestyle and a decrease in proteinuria are also beneficial to the management of DKD (Nangaku et al., 2005). There are several therapies for DKD that are being studied in phase 3+ clinical trials, including endothelin-1 receptor A antagonists, mineralocorticoid receptor antagonists, TGF-β inhibitors, phosphodiesterase inhibitors, and 5-Hydroxytryptamine 2a receptor antagonists (Doshi and Friedman, 2017). Accumulating evidence suggests that mitochondrial dysfunction plays a critical role in the progression of DKD. A variety of novel mitochondria-targeted approaches is currently in development to prevent and treat DKD (a summary is in Table 1; Yang et al., 2017).

**TABLE 1:** Mitochondrial-targeted therapeutics in experimental models of DKD.

Mitochondrial-targeted therapeutics in experimental models of DKD			
Mitochondrial-targeted agent	Pathway or Category	Major Findings	References
Empagliflozin	AMPK/SP1/PGAM5 pathway	Alleviated mitochondrial fission	Khaminets et al., 2016
Berberine	Mitochondrial dysfunction pathway	Inhibited mitochondrial fragmentation and dysfunction	Kazlauskaite et al., 2014
Mdivi1	Inhibitor of Drp1	Alleviated mitochondrial fission and rescued key pathologic features of DKD	Kaushik and Cuervo, 2018
MitoQ	Nrf2/PINK1 pathway	Inhibited DRP1, promoted MFN2_:_ restored mitophagy	Puigserver et al., 1998
SRT1720	SIRTl-PGC-1α axis	Induced mitochondrial biogenesis	Youle and van der Bliek, 2012
Resveratrol	AMPK-SIRTl-PGC-1α axis	Induced mitochondrial biogenesis	Zhan et al., 2018
rPGRN	PGRN-Sirt1-PGC-1α/FoxO1	Enhanced mitochondrial biogenesis and mitophagy	Zhan et al., 2015
TEPP-46	A small-molecule PKM2 activator	Induced mitochondrial biogenesis	Zhong et al., 2018
SS-31	A mitochondria-targeted tetrapeptide	Reduced glomerular hypertrophy, mesangial expansion and decreased apoptosis	Zhou et al., 2019
Fenofibrate	PPARα modulator	Restored mitochondrial fatty acid β-oxidation	Zhu et al., 2013

Inhibition of mitochondrial fragmentation has been demonstrated to protect against DKD. Empagliflozin is a pharmacologic intervention targeting sodium-glucose co-transporter 2 (SGLT2) that has been widely used as a new treatment option for DM (Perkovic et al., 2019). Clinical trials have observed encouraging effects of SGLT2 inhibitors on DKD. The extra-renal protective effect of SGLT2 inhibitors confirms beyond the control of hyperglycemia. Empagliflozin could remarkably reduce the expression of PGAM5 by activating AMPK, then alleviating mitochondrial fission to treat DKD (Liu et al., 2020). DRP1 mediates the process of Mitochondrial Fission. Berberine (BBR) inhibits DRP1-mediated mitochondrial fission then protects renal cells (Qin et al., 2019). Mdivi1, the inhibitor of DRP1, is sufficient to block mitochondrial fission and improve DKD (Ayanga et al., 2016).

Enhancement of the removal of damaged and dysfunctional mitochondria by mitophagy in kidney tubular cells is also beneficial for the treatment of DKD (Bhatia and Choi, 2019). MitoQ can reverse the deficient mitophagy, which up-regulates PINK1 and Parkin expression and inhibit mitochondrial ROS production in DKD (Xiao et al., 2017).

Stimulation of mitochondrial biogenesis is another attractive strategy for preventing DKD. The role of PGC-1α is essential in controlling the process of mitochondrial biogenesis (Scarpulla, 2011), pharmacological activation of PGC1α serves as a novel and potential approach to improve this disease of DKD. Enhancement of mitochondrial biogenesis using AMPK activators and sirtuin 1 activators (Higashida et al., 2013; Hong et al., 2018; Zhong et al., 2018) [SRT1720 and resveratrol (Higashida et al., 2013; Kitada and Koya, 2013)], which can increase PGC-1α expression and protect against high glucose-mediated mitochondrial injury. In addition, Recombinant human progranulin (rPGRN) attenuated high glucose-induced mitochondrial dysfunction by facilitating mitophagy and mitochondrial biogenesis (Zhou et al., 2019). TEPP-46, a small-molecule PKM2 activator, can protect against DKD by inhibiting the production of toxic glucose metabolites and increasing PGC-1α to restore mitochondrial function (Qi et al., 2017).

Moreover, SS-31, a mitochondria-targeted tetrapeptide, has been studied in models of DKD, which can scavenge ROS, decrease mesangial expansion, and tubular apoptosis (Hou et al., 2016). Fenofibrate reduced albuminuria and slowed GFR impairment and restored mitochondrial fatty acid β-oxidation in patients with DKD, which is a PPARα activator (Davis et al., 2011).

## Discussion and Conclusion

**FIGURE 2:**
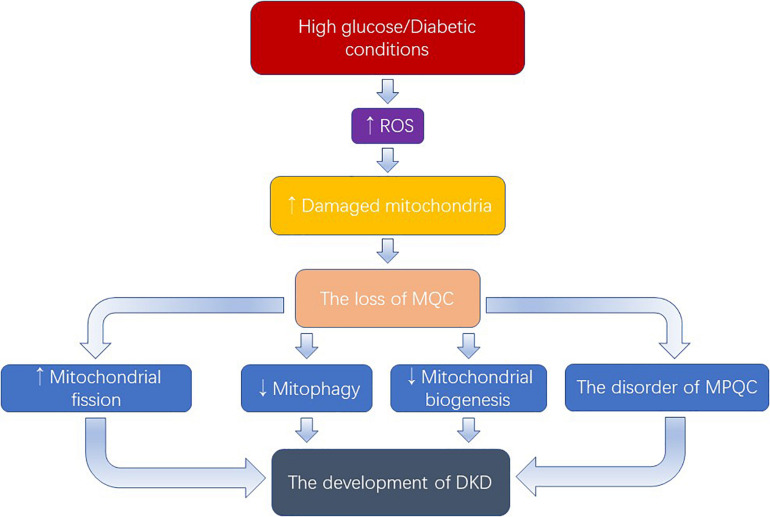
Excess ROS produced by the activation of high glucose causes damage to mitochondria. When the number of damaged mitochondria overwhelms the mitophagy process, the mitochondrial quality control will be damaged. Owning to the rich mitochondrial in the kidney, the loss of MQC exerts an important role in the development of DKD. MQC: mitochondrial quality control; MPQC: mitochondrial protein quality control.

Hyperglycemia, inducing the overproduction of ROS which damages mitochondria, plays a key role in the progression of DKD (Figure 2). As discussed above, we have known that excessive mitochondrial fission, dysregulation of mitophagy, defective mitochondrial biogenesis, and the disorder of mitochondrial protein quality control mechanism occurred in DKD, as well as the therapeutic potential of targeting mitochondrial quality control mechanisms. In addition, the mechanism of mitochondrial quality control is being further improved. Recently, Yu et al discovered a new mechanism called mitocytosis, which is a migrasome-mediated mitochondrial quality control process, maintaining mitochondrial membrane potential and viability in neutrophils (Jiao et al., 2021). However, several questions remain.

First, although emerging evidence suggests that the disorder of mitochondrial protein quality control plays a part in DKD, their precise roles in these pathological conditions and the molecular machinery that is involved remain largely unknown. In the future, we need to further explore the mechanism of mitochondrial protein quality control in the development of DKD, then providing a new theoretical basis for the treatment or delay of this disease.

Second, healthy and functional mitochondria operating by mitochondrial quality control mechanisms are interconnected networks. Therefore, any changes in the quality control mechanisms will affect other mechanisms, which in turn affect the entire system. Take an instance, mitochondrial fission is necessary for mitophagy and also regulates mitochondrial biogenesis. So, the pharmacological inhibition of mitochondrial fission may also damage the biogenesis of mitochondria and mitophagy.

Finally, a large number of studies that provide a new therapy for DKD targeting mitochondrial quality control mechanisms are at the stage of cell and animal models at present. A large amount of experimental research evidence is still needed for clinical application, which faces big challenges.

## Author Contributions

LH conceived of the topic for this review. WD wrote the manuscript. All authors provided intellectual input to the editorial.

## Conflict of Interest

The authors declare that the research was conducted in the absence of any commercial or financial relationships that could be construed as a potential conflict of interest.

## Publisher’s Note

All claims expressed in this article are solely those of the authors and do not necessarily represent those of their affiliated organizations, or those of the publisher, the editors and the reviewers. Any product that may be evaluated in this article, or claim that may be made by its manufacturer, is not guaranteed or endorsed by the publisher.
